# Optimization of Ibuprofen Route and Dosage to Enhance Protein-Bound Uremic Toxin Clearance During Hemodialysis

**DOI:** 10.3390/toxins18010037

**Published:** 2026-01-11

**Authors:** Víctor Joaquín Escudero-Saiz, Elena Cuadrado-Payán, María Rodríguez-García, Gregori Casals, Lida María Rodas, Néstor Fontseré, María del Carmen Salgado, Carla Bastida, Nayra Rico, José Jesús Broseta, Francisco Maduell

**Affiliations:** 1 Nephrology and Renal Transplantation, Hospital Clínic de Barcelona, 08036 Barcelona, Spain; victorescuderosaiz4@gmail.com (V.J.E.-S.); ecuadrado@clinic.cat (E.C.-P.); lmrodas@clinic.cat (L.M.R.); fontsere@clinic.cat (N.F.); jjbroseta@clinic.cat (J.J.B.); 2 Medicine Department, Faculty of Medicine and Health Sciences, University of Barcelona, 08036 Barcelona, Spain; 3 Biochemistry and Molecular Genetics Department—CDB, Hospital Clínic de Barcelona, 08036 Barcelona, Spain; mrodriguezg@clinic.cat (M.R.-G.); casals@clinic.cat (G.C.); salgado@clinic.cat (M.d.C.S.); nrico@clinic.cat (N.R.); 4 Pharmacy Department, University of Barcelona, 08036 Barcelona, Spain; cbastida@clinic.cat

**Keywords:** arterial line infusion, displacer, ibuprofen, indoxyl sulphate, online hemodiafiltration, p-cresyl sulphate, protein-bound uremic toxins

## Abstract

Protein-bound uremic toxins (PBUT), particularly indoxyl sulphate (IS) and p-cresyl sulphate (pCS), are poorly removed by conventional haemodialysis because of their strong albumin binding. These toxins are associated with cardiovascular morbidity and mortality in haemodialysis patients. Displacer molecules such as ibuprofen enhance PBUT clearance by competing for albumin-binding sites, but the optimal dose and route of administration remain unclear. The aim of this study was to evaluate the effect of different ibuprofen doses, infusion durations, and routes of administration on the removal of IS and pCS during on-line hemodiafiltration (OL-HDF). In this prospective, single-centre, crossover study, 21 chronic haemodialysis patients receiving intradialytic analgesia underwent nine OL-HDF sessions. Ibuprofen was administered at two doses (400 or 800 mg) either in the arterial pre-filter line (infusion over 1 h, 2 h, or 3 h) or in the venous post-filter line (30 min). Reduction ratios (RR) of total IS and pCS were determined by LC-MS and corrected for haemoconcentration. Statistical analysis included repeated-measures ANOVA with post-hoc testing. Baseline RR for IS and pCS were 53.7 ± 9.9% and 47.1 ± 10.9%, respectively. The highest RR was achieved with 800 mg ibuprofen infused via the arterial line over 2 h (IS: 60.8 ± 8.6%; pCS: 57.8 ± 9.7%). All arterial-line 800 mg regimens and the 3-h 400 mg infusion significantly improved pCS clearance versus baseline; IS clearance improved significantly only with arterial-line 800 mg regimens and with the 400 mg 3-h infusion. Infusion rate (1–3 h) had no significant effect on RR within the same dose group. Pain scores decreased significantly after dialysis regardless of ibuprofen regimen. Arterial-line administration of ibuprofen enhances total IS and pCS removal during OL-HDF, with higher doses yielding greater clearance. Prolonged low-dose infusion appears similarly effective for pCS and may reduce systemic exposure, potentially lowering toxicity risk. These findings support the arterial line as the preferred route for displacer administration in clinical practice.

## 1. Introduction

Protein-bound uremic toxins (PBUT) are at the forefront of haemodialysis innovation. There are several molecules inside this group, but indoxyl sulphate (IS) and p-cresyl sulphate (pCS) are two of the most studied [1]. These toxins are generated through an initial metabolism by the gut microbiota, followed by hepatic metabolism [2]. They are considered among the most toxic uremic toxins known by the European Uremic Toxins (EUTox) working group [3]. In fact, pCS is considered the most toxic one with the highest toxicity score with current experimental and clinical data, affecting up to seven different organs [4].

PBUT are low molecular-weight toxins whose removal is under 60% with standard haemodialysis techniques [4,5] due to their high-affinity binding to albumin [6]. Among PBUTs, 3-carboxy-4-methyl-5-propyl-2-furanpropanoic acid (CMPF) exhibits the highest reported protein-binding capacity, with values ranging from 99% to 100% [7] while IS and pCS reach an affinity about 95% [6,7]. IS and pCS have been related to cardiovascular morbidity and mortality in haemodialysis patients [8,9,10].

Nowadays, some strategies are under development for increasing PBUT removal [1]. Among these, we can find the improvement in adsorptive capability of modern dialysis membranes [11], the use of molecules that compete with PBUT for their albumin binding site [12] or even the addition of displacers inside the blood-contacting surface of the dialysis membrane [13]. However, there is only clinical information about the use of displacers during the haemodialysis session in humans with ibuprofen [14,15]. More recently, pre-dialysis administration of loop diuretics has shown an increase in PBUT removal during dialysis [16].

In a previous work, we examined the efficacy of ibuprofen in comparison with other analgesics with their administration in the arterial line [15]. Although it is reasonable to think that the arterial dialysis line would be the most effective route of displacer’s administration, the posology of the displacer molecule must be assessed. The aim of this study **was** to determine the best route and dosage of ibuprofen, administered during online hemodiafiltration (HDF) sessions, that maximizes PBUT clearance.

## 2. Results

### 2.1. Baseline Characteristics

A total of 21 patients were included with a mean age of 76.7 ± 15.8 years (66.7% women) (range 21–91) on a regular haemodialysis program for 52.3 ± 63.9 months (range 8–274). Vascular accesses were 11 native arterio-venous fistula (52.4%) and 10 tunnelled central venous catheter (47.6%). The anticoagulation used was low-molecular weight heparin in 11 patients (52.4%) and heparin sodium in 8 patients (38.1%); the remaining 2 patients had systemic anticoagulation therapy. Underlying renal diseases were nephroangiosclerosis (7 patients), diabetic nephropathy (2 patients), urologic aetiology (2 patients), interstitial nephritis (2 patients), and unknown aetiology (3 patients).

### 2.2. Pain Characteristics

Pain causes were as follows: 10 mechanic (47.6%), 3 neck-pain (14.3%), 2 joint-pain (9.5%), 2 headache (9.5%), 1 hydrocele (4.8%), 1 pain related to punction of nAVF (4.8%), 1 distal neuropathy (4.8%), and 1 digital ischemia (4.8%).

The lowest pain values were observed in the control session (Table 1). The mean pain among the rest sessions was 3.5 ± 0.4 points and 0.9 ± 0.2 points before and after dialysis, respectively. A significant reduction in pain was observed post-dialysis, irrespective of the ibuprofen regimen administered (Table 1).

### 2.3. Dialysis Parameters

Dialysis parameters of each session are shown in Table 2. Dialysis parameters remained consistent across all study arms, with no statistically significant variations detected.

### 2.4. Protein-Bound Uremic Toxins Removal

#### 2.4.1. Every Intervention

Pre- and post-dialysis IS and pCS values are shown in Table 3, as well as their reduction ratios (RR). Baseline RR was 53.7 ± 9.9 and 47.1 ± 10.9 for IS and pCS, respectively. The highest RR observed was seen with the administration of 800 mg of ibuprofen during two hours with 60.8 ± 8.6 and 57.8 ± 9.7 for IS and pCS, respectively.

**Figure 1:**
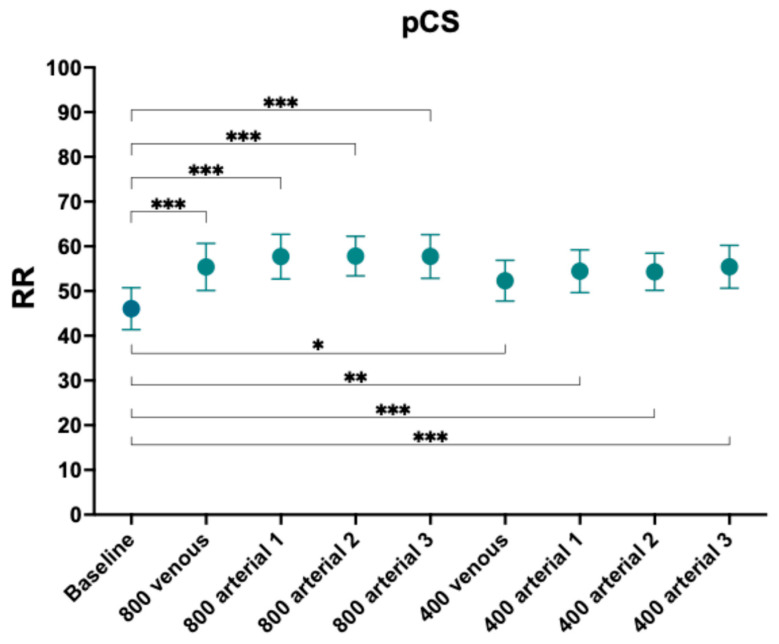
Reduction ratios of p-cresyl sulphate. Comparison with baseline. *, *p*-value < 0.05, **, *p*-value < 0.005; ***, *p*-value < 0.001.

RR for pCS increased significantly irrespective of the posology or dose of ibuprofen compared to baseline (Figure 1 and Table 4).

**Figure 2:**
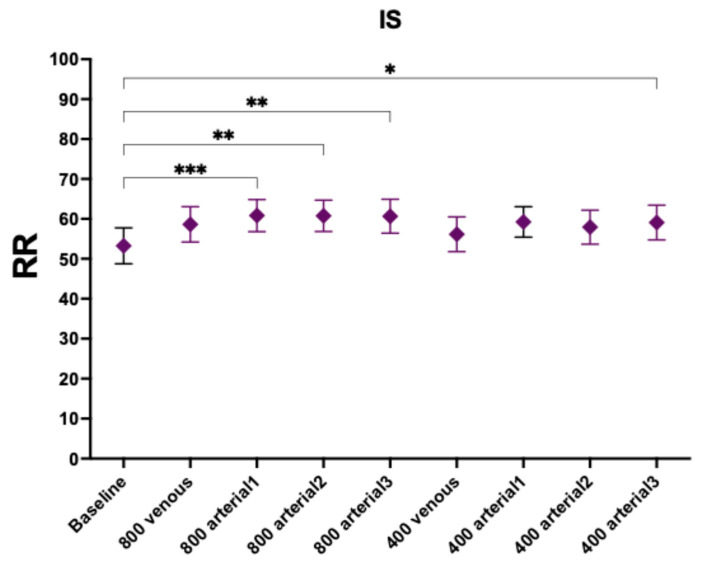
Reduction ratios of indoxyl sulphate. Comparison with baseline. *, *p*-value < 0.05; **, *p*-value < 0.005; ***, *p*-value < 0.001.

RR for IS increased significantly for the 800 mg administration in arterial line uninfluenced by the time of administration (Figure 2 and Table 4). However, none of the dosing regimens administered in the venous line increased the RR of IS. Regarding the lower-dose arms (400 mg), only the route with the longest duration achieved statistical significance (arterial 3-h 400 mg, *p*-value 0.0319).

#### 2.4.2. Time Effect

When analyzing the interventions with the same ibuprofen dose, there were no statistically significant differences detected according to the administration rate.

#### 2.4.3. Dose Effect

**Figure 3:**
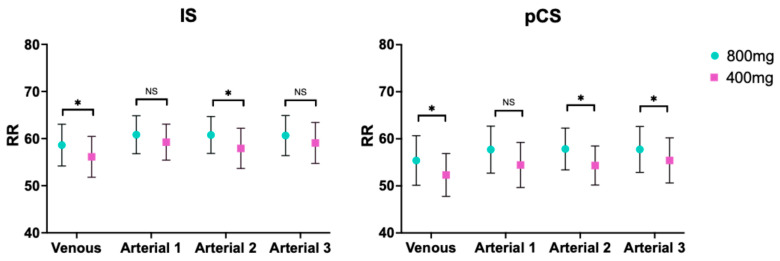
Comparison between doses for indoxyl sulphate and p-cresyl sulphate. RR, reduction ratio; IS, indoxyl sulphate (**left**); pCS, p-cresyl sulphate (**right**). *, *p*-value < 0.05; NS, non-significant.

When comparing the same route and rate of administration between the two doses (800 mg vs. 400 mg), there were differences in both venous route (*p*-value 0.0408 and *p*-value 0.0168, respectively) and in the 2-h administration (*p*-value 0.0183 and *p*-value 0.0132, respectively) for IS and pCS, respectively. Regarding the longest time of administration, there were only statistically significant differences for pCS (*p*-value 0.0459). Last, the 1-h administration did not show differences (Table 5 and Figure 3).

## 3. Discussion

This work demonstrates that the administration of ibuprofen through the arterial haemodialysis line is the most effective approach to enhance IS and pCS removal during haemodialysis. In addition, prolonged and slower displacer administration achieves comparable improvements in IS and pCS clearance, which may represent an advantage by potentially reducing the risk of adverse effects related to the displacer. Additionally, our findings also indicate that lower ibuprofen doses are sufficient to significantly increase pCS removal, while higher displacer concentrations result in greater overall clearance of both toxins.

PBUTs bind to albumin at Sudlow binding sites I and II with different affinities, being approximately 95% for IS and pCS [6]. Their toxicity in various systems has been demonstrated [4], with pCS being regarded as the most toxic uremic toxin known [3]. Both pCS and IS are considered endothelial toxins [17] and have been associated with increased cardiovascular morbidity and mortality rates in patients undergoing haemodialysis [8,9,10] and in patients with non-dialysis chronic kidney disease (CKD) [18,19]. More recently, kynurenines, another PBUT group, have been related to non-atheromatous cardiovascular events in a national and observational study in patients with non-dialysis CKD [20].

Because of their high-affinity to albumin their removal during a conventional high-flux haemodialysis [21,22], expanded-haemodialysis [23] or post-dilutional HDF [21,24,25] is less than 50% and 55% for IS and pCS, respectively, despite their low molecular-weight [5]. Longer haemodialysis sessions show the highest reduction ratios in clinical scenarios with 66% for IS and 59% for pCS with high-flux haemodialysis with high KoA membranes [26] and 60% and 52% for IS and pCS, respectively, with OL-HDF [21]. For all these reasons, PBUT removal is at the forefront of dialysis investigation due to their variety of toxic effects.

Some strategies have been studied for improving PBUT removal during the haemodialysis session in the last years [1]. Among these, the use of molecules which bind and compete to the same albumin-site as PBUT are the most available until these days [12]. Different molecules have been described and tested in vitro, such as tryptophan, furosemide, and ibuprofen [27,28] with the most powerful results observed with ibuprofen or its combination with furosemide in vitro [28]. Specifically, some studies have shown the displacer capability of ibuprofen in vitro, which also binds to Sudlow site II and therefore increases the free fraction of IS and pCS due to its higher affinity for albumin [27,28].

In murine models, other molecules such as salvianolic acids have been used in an in-vivo murine model with an increased effect of 119.55% and 127.56% of IS and pCS removal, respectively, due to an allosteric effect instead of direct competition [29]. Besides, intravenous lipid emulsions (Intralipid^TM^, Fresenius KABI SSPC, Jiangsu, China), which release free-fatty acids through plasmatic lipoprotein lipases metabolism, have also been shown to enhance the clearance of different PBUT, such as IS, pCS, and CMPF, the latter for the first time [30]. Nonetheless, data is scarce in the clinical scenario. The first work that assessed the displacer capability of ibuprofen in vivo were Madero et al., who found a relative increase in IS and pCS removal of 237% and 239%, respectively, with the administration of 800 mg of ibuprofen for 40 min in the pre-filter arterial line in 18 haemodialysis patients [14]. In a more recent work, pre-dialysis orally administration of loop-diuretics showed an increased effect on pCS and IS removal in 17 anuric patients on regular haemodialysis [16].

Our findings support the use of any displacer molecule in the arterial pre-filter line over other places during the haemodialysis session, as it reaches the highest increase of IS and pCS removal up to 61% and 58%, respectively, for 800 mg of ibuprofen compared to 59% and 55% with the post-filter venous administration (equivalent to systemic administration). Regarding the lower 400 mg dose, IS and pCS removal reaches 59% and 54%, respectively, compared to 56% and 52% of the venous administration. However, these differences do not reach statistical significancy in the multiple comparison model. Nonetheless, the only strategies that maintain their statistically difference with baseline are all the 800 mg and the longest 400 mg (3-h) arterial administrations, without statistically differences with the venous post-filter administration for IS (Table 4). For pCS, every strategy was successful statistically.

We also aimed to evaluate the efficacy depending on the time of administration. There were no differences between different displacer infusion rates for the same subgroups (400 mg and 800 mg, separately). This lack of difference might be clinically meaningful and potentially advantageous. One of the most critical considerations in the use of displacer such as ibuprofen in haemodialysis patients is its safety profile and spectrum of adverse events. Given that ibuprofen is a dialyzable molecule because its molecular characteristics [31], its slower and more prolonged administration through the pre-filter arterial line would be expected to reduce its systemic bioavailability as a result of both dialytic removal and systemic metabolism. This approach may therefore help to mitigate safety concerns while maintaining efficacy. In fact, a recent mathematical in vitro haemodialysis model shows that only 38% of an 800 mg ibuprofen dose is detected after a 4-h dialysis session [27]. Therefore, a more prolonged and slower administration of ibuprofen could reduce the likelihood of adverse events while simultaneously increasing the clearance of PBUT. However, further studies specifically addressing the hypothetical differences in serum ibuprofen concentration are required as the dialyzability of ibuprofen depends on its free fraction.

Last but not least, it is worth highlighting that lower dose displacer infusion reached statistically differences of RR of pCS compared to baseline, while only the 3-h infusion reached significant differences for IS removal. It might be explained for the fact of the multiple competition between all PBUT and ibuprofen to Sudlow site II. Nonetheless, the higher the ibuprofen dose, the higher PBUT removal, as evidenced by the observation that the clearance of IS and pCS with 800 mg is statistically superior to its equivalent infusion at half the dose (Figure 3). Thereby, lower displacer dose may reduce the risk of toxicity at the same time of increasing PBUT removal. Whether a reduced likelihood of adverse events associated with ibuprofen administration in hemodialysis patients can be achieved through a more prolonged and slower infusion rate or through lower ibuprofen concentrations should be specifically evaluated in future studies.

Notwithstanding, to date, no specific thresholds have been established at which toxicity related to IS and pCS becomes evident. However, the risk of cardiovascular mortality associated with these toxins correlates directly and continuously with increasing serum concentrations, both in patients with chronic kidney disease undergoing haemodialysis and in those not receiving dialysis [32]. Consequently, further studies investigating molecular, endothelial, and hard outcomes concerning the impact of reducing PBUTs in hemodialysis patients are required.

This work faces certain limitations. First, the relatively small sample size restricts, and the single-center design may limit its generalizability. In addition, the necessity of adjusting for multiples comparison through stringent statistical thresholds might have underpowered the detection of smaller, albeit clinically relevant, differences between infusion rates. Second, we only assessed IS and pCS, limiting the extrapolation of these results to other not measured PBUT. Third, serum ibuprofen concentrations were not measured, preventing experimental confirmation of the predictions derived from the above-mentioned mathematical model. However, this is the first work that compares different displacer administration sources to evaluate their efficacy in increasing PBUT removal in patients on regular haemodialysis treatment.

In conclusion, the administration of ibuprofen into the arterial line (pre-filter) during the hemodialysis session significantly increases PBUT clearance compared to other sites. Lower doses of ibuprofen are significantly effective in enhancing PBUT removal. Slower and more prolonged infusions are equally effective in increasing PBUT clearance and may offer a better safety profile. More robust studies specifically addressing these issues are needed for determining the best displacer posology during hemodialysis.

## 4. Materials and Methods

### 4.1. Inclusion and Exclusion Criteria for the Sample

This prospective, single-center study included 21 patients with a prescription of intradialytic treatment for pain from our dialysis unit. The patients included did not present any exclusion criteria: presence of residual kidney function, previous history of gastrointestinal bleeding, known adverse reaction to ibuprofen and treatment with other PBUT displacing molecules (loop diuretics, tryptophan, salvianolic acids, or intravenous lipid emulsions).

### 4.2. Data Collection and Procedure

The study followed a prospective, single-center, crossover design. Each patient underwent nine OL-HDF sessions under different ibuprofen administration regimens. Baseline demographic and clinical characteristics were collected at study entry, including age, sex, dialysis vintage, and relevant comorbidities.

The prescribed dialysis parameters were post-dilution HDF, bicarbonate buffer, dialysate flow (Qd) 400 mL/min, blood flow (Qb) 421.4 ± 25.4 mL/min (range 400–450), and dialysis time 294.3 ± 12.1 min (range 270–300). Dialyzer membranes were 15 FX CorDiax 60^®^ (71.4%) (Fresenius, Bad Homburg, Germany), 3 Clearum^®^ HS (14.3%) (Bellco S.r.l., Mirandola, Italy), 2 CorAL 60^®^ (9.5%) (Fresenius, Bad Homburg, Germany), and 1 Solacea^®^ (Nipro Medical Corporation, Osaka, Japan). Net fluid removal was set individually, depending on the patient’s clinical needs. Fresenius 5008 Cordiax or 6008 CAREsystem (Fresenius, Bad Homburg, Germany) dialysis monitors were used. The variables collected in each session were as follows: real duration, dialyzer, Qb, recirculation index measured by the temperature module, arterial, venous, and transmembrane (TMP) pressures, initial and final haematocrit automatically measured by the BVM^®^ biosensor (Fresenius, Bad Homburg, Germany), initial and final body weights, volume of blood processed, and replacement volume.

Each patient underwent nine dialysis sessions with routine dialysis parameters in which only the route and dosage of ibuprofen was modified. The different sessions in which ibuprofen was administered were randomized. There were two presentations of ibuprofen (800 mg and 400 mg diluted in 250 mL of NaCl 0.9%) which were administered with different infusion rates depending on the source of intra-dialysis administration. For the arterial line source, there were three different rates: one hour, two hours and three hours, while for the venous route there was only 30 min rate of infusion.

Blood and dialysis fluid samples for analyses were taken from each patient in the same dialysis session of the week.

### 4.3. Research Instrument

Protein-bound uremic toxins (PBUT), p-cresyl sulphate (MW 108) and indoxyl sulphate (MW 213), were corrected for the degree of the haemoconcentration and the volume of distribution (approximate extracellular volume) according to Bergström and Wehle [33]. Total IS and pCS were measured in serum before and after each OL-HDF session using liquid chromatography-mass spectrometry (LC-MS) [34]. Total IS and pCS values were measured in ng/mL and reduction ratios were calculated. More information about the biochemical methodology can be found in our previous paper [34]. Pain was assessed using the WHO visual analogue scale (VAS) before and after each session. Adverse events and treatment tolerance were recorded using structured clinical report forms and patient medical records.

### 4.4. Ethical Considerations

All patients provided informed consent. The study was approved by the local ethic committee (HCB/2024/0173) and was conducted according to the principles of the Declaration of Helsinki.

### 4.5. Statistical Analysis

Statistical analyses and graphics were performed using GraphPad Prism version 10.2.3 (GraphPad Software, Boston, MA, USA). The results are expressed as the arithmetic mean ± standard deviation. For the analysis of the statistical significance of quantitative parameters, the Student’s *t*-test for paired data was used for assessing the analgesic effect. One-way ANOVA for repeated measurements followed by Dunnet’s post-hoc comparisons tests was applied to compare each intervention with the baseline condition, whereas Tukey’s post-hoc test was used to assess pairwise differences among all interventions. A *p* < 0.05 was considered statistically significant.

## Figures and Tables

**Table 1:** Values of WHO visual analogue scale before and after each session.

Posology	Pre-Dialysis	Post-Dialysis	*p*-Value
Baseline	1.1 ± 2.0	0.5 ± 1.2	0.102
800 mg—venous	3.1 ± 2.3	0.7 ± 1.0	<0.001
800 mg—arterial 1	4.1 ± 3.1	1.2 ± 1.9	<0.001
800 mg—arterial 2	3.4 ± 2.7	1.1 ± 1.6	<0.001
800 mg—arterial 3	3.5 ± 2.6	0.7 ± 1.2	<0.001
400 mg—venous	3.0 ± 2.9	0.5 ± 1.3	<0.001
400 mg—arterial 1	3.2 ± 2.6	1 ± 1.9	<0.001
400 mg—arterial 2	3.9 ± 3.0	0.9 ± 1.5	<0.001
400 mg—arterial 3	3.8 ± 2.4	0.9 ± 1.4	<0.001

*p*-value of Student’s *t*-test for paired data are shown.

**Table 2:** Dialysis parameters.

	Baseline	400 mg Venous	400 mg Arterial 1	400 mg Arterial 2	400 mg Arterial 3	800 mg Venous	800 mg Arterial 1	800 mg Arterial 2	800 mg Arterial 3
Total blood (L)	116.9 ± 12.1	117.4 ± 13.8	118.2 ± 12.5	118.3 ± 13.2	115.1 ± 14.7	118.3 ± 13.3	116.1 ± 13.1	117.5 ± 12.2	120.2 ± 12.4
Substitution volume (L)	30.3 ± 7.3	29.7 ± 4.5	29.9 ± 5.5	30.9 ± 6.2	28.3 ± 5.3	30.7 ± 5.7	30.2 ± 5.9	29.9 ± 5.5	30.0 ± 6.3
Initial weight (Kg)	66.2 ± 11.8	66.4 ± 11.5	66.2 ± 11.7	66.7 ± 11.7	66.4 ± 11.8	67.6 ± 12.1	66.3 ± 11.3	66.3 ± 11.4	66.2 ± 11.6
Final weight (Kg)	64.6 ± 11.6	64.3 ± 11.7	64.4 ± 11.7	64.5 ± 11.8	64.3 ± 11.8	65.6 ± 12.3	64.6 ± 11.3	64.4 ± 11.6	64.4 ± 11.9
Weight gain (Kg)	1.6 ± 0.9	2.1 ± 0.8	1.7 ± 0.5	2.2 ± 0.9	2.1 ± 0.8	2.0 ± 0.7	1.7 ± 0.7	1.9 ± 0.7	1.8 ± 0.8
Real time (min)	288.5 ± 11.8	286.1 ± 13.8	285.8 ± 14.4	286.0 ± 13.5	285.9 ± 13.0	288.0 ± 10.3	286.7 ± 13.2	286.9 ± 13.2	287.2 ± 11.9
Haematocrit pre (%)	30.3 ± 5.0	29.5 ± 4.4	30.1 ± 4.7	28.8 ± 5.0	29.7 ± 4.7	30.1 ± 6.0	29.1 ± 5.2	30.8 ± 5.6	30.5 ± 5.2
Haematocrit post (%)	34.3 ± 5.4	33.8 ± 5.7	34.2 ± 5.5	33.3 ± 6.3	34.8 ± 5.4	34.6 ± 6.0	32.8 ± 5.6	35.0 ± 6.7	34.9 ± 6.5
KT (L)	69.2 ± 8.7	68.8 ± 8.3	69.0 ± 7.9	70.2 ± 9.8	67.5 ± 8.4	69.6 ± 7.3	68.8 ± 6.6	68.7 ± 7.7	68.2 ± 7.6
Recirculation (%)	15.4 ± 4.6	13.9 ± 3.1	14.6 ± 3.6	14.5 ± 4.2	15.1 ± 6.3	14.6 ± 5.6	14.8 ± 3.3	15.5 ± 5.3	15.7 ± 4.8
Arterial Pressure (mmHg)	−233 ± 35	−222 ± 23	−222 ± 29	−226 ± 26	−221 ± 35	−226 ± 29	−229 ± 40	−226 ± 38	−224 ± 31
Venous Pressure (mmHg)	187 ± 36	182 ± 31	182 ± 39	182 ± 28	185 ± 48	182 ± 27	183 ± 37	191 ± 25	183 ± 33
TMP (mmHg)	150 ± 38	159 ± 37	150 ± 32	167 ± 43	152 ± 42	153 ± 52	165 ± 45	152 ± 48	156 ± 46

L, litres; Kg, kilograms; min, minutes; pre, pre-dialysis; post, post-dialysis; %, percentage; mmHg, millimetres of mercury; TMP, transmembrane pressure.

**Table 3:** Indoxyl sulphate and p-cresyl sulphate values and reduction ratios.

Posology	IS			pCS		
	Pre-Dialysis	Post-Dialysis	RR (%)	Pre-Dialysis	Post-Dialysis	RR (%)
Baseline	25,908 ± 16,120	11,748 ± 6970	53.7 ± 9.9	35,529 ± 13,656	19,610 ± 8479	47.1 ± 10.9
800 mg venous	25,697 ± 13,286	10,169 ± 4819	58.6 ± 9.7	36,418 ± 15,181	15,847 ± 6628	55.4 ± 11.6
800 mg arterial 1	25,634 ± 15,258	9522 ± 5195	60.8 ± 8.6	39,222 ± 17,488	16,518 ± 7419	57.7 ± 11.0
800 mg arterial 2	25,204 ± 14,676	9577 ± 5375	60.8 ± 8.6	34,320 ± 13,135	14,632 ± 7140	57.8 ± 9.7
800 mg arterial 3	25,582 ± 13,411	9432 ± 4451	60.7 ± 9.3	36,419 ± 15,175	15,076 ± 6577	57.7 ± 10.7
400 mg venous	23,962 ± 11,949	10,068 ± 4506	56.3 ± 9.7	34,762 ± 17,715	16,812 ± 9576	52.3 ± 10.0
400 mg arterial 1	23,633 ± 13,605	9269 ± 5077	59.2 ± 8.4	35,283 ± 12,269	15,782 ± 6103	54.3 ± 10.4
400 mg arterial 2	23,358 ± 11,311	9195 ± 3663	57.9 ± 9.4	34,488 ± 13,305	15,534 ± 6745	54.3 ± 9.1
400 mg arterial 3	25,210 ± 12,813	9995 ± 5010	59.1 ± 9.6	36,877 ± 12,685	16,297 ± 6440	55.4 ± 10.5

IS, indoxyl-sulphate; pCS, p-cresyl sulphate; RR, reduction ratio. IS and pCS values are measured in ng/mL.

**Table 4:** Comparison of reduction ratio of indoxyl sulphate and p-cresyl sulphate with baseline.

Comparison	IS			pCS		
	Mean Difference	CI of Difference	Adjusted *p*-Value	Mean Difference	CI of Difference	Adjusted *p*-Value
Baseline vs. 800 venous	−5.367	−10.79 to 0.05391	0.0530	−9.338	−14.91 to −3.770	**0.0006**
Baseline vs. 800 arterial1	−7.571	−12.25 to −2.889	**0.0009**	−11.65	−16.92 to −6.389	**<0.0001**
Baseline vs. 800 arterial2	−7.510	−12.44 to −2.575	**0.0018**	−11.79	−16.15 to −7.423	**<0.0001**
Baseline vs. 800 arterial3	−7.395	−12.86 to −1.930	**0.0053**	−11.69	−17.35 to −6.032	**<0.0001**
Baseline vs. 400 venous	−2.876	−8.329 to 2.577	0.5366	−6.271	−11.55 to −0.9955	**0.0155**
Baseline vs. 400 arterial1	−5.990	−12.40 to 0.4143	0.0732	−8.386	−15.06 to −1.716	**0.0100**
Baseline vs. 400 arterial2	−4.667	−10.04 to 0.7074	0.1072	−8.271	−12.74 to −3.805	**0.0002**
Baseline vs. 400 arterial3	−5.824	−11.25 to −0.3998	**0.0319**	−9.367	−14.81 to −3.919	**0.0005**

One-way ANOVA with Geisser–Greenhouse’s correction for repeated measures with Dunnet’s correction post-hoc was used. CI, confidence interval; IS, indoxyl sulphate; pCS, p-cresyl sulphate.

**Table 5:** Specific comparison between dosages.

Comparison	IS			pCS		
	Mean of Differences	95% CI	*p* Value	Mean of Differences	95% CI	*p* Value
800 venous vs. 400 venous	−2.490	−5.323 to 0.3419	**0.0408**	−3.067	−5.871 to −0.2622	**0.0168**
800 arterial 1 vs. 400 arterial 1	−1.581	−4.640 to 1.478	0.1469	−3.267	−7.746 to 1.213	0.0719
800 arterial 2 vs. 400 arterial 2	−2.843	−5.489 to −0.1965	**0.0183**	−3.514	−6.571 to −0.4576	**0.0132**
800 arterial 3 vs. 400 arterial 3	−1.571	−3.897 to 0.7539	0.0870	−2.324	−5.061 to 0.4135	**0.0459**

One-tailed T-Student test for repeated measures was used.

## Data Availability

The original contributions presented in this study are included in the article. Further inquiries can be directed to the corresponding author(s).
